# Stigma Barriers of Mental Health in Iran: A Qualitative Study by Stakeholders of Mental Health

**Published:** 2017-07

**Authors:** Arsia Taghva, Zahra Farsi, Yavar Javanmard, Afsaneh Atashi, Ahmad Hajebi, Mojgan Khademi

**Affiliations:** 1 AJA University of Medical Sciences, Tehran, Iran.; 2 Community Health Department, Faculty of Nursing, AJA University of Medical Sciences, Tehran, Iran.; 3 Faculty of Psychology, Bangalore University, Bangalore, India.; 4 Addiction and High Risk Behavior, Tehran Institute of Psychiatry, Faculty of Behavioral Sciences and Mental Health, Iran University of Medical Sciences (IUMS), Tehran, Iran.; 5 Department of Child Psychiatry, Shahid Beheshti University of Medical Sciences, Tehran**, **Iran**. **

**Keywords:** *Stigma Reduction*, *Mental Disorders*, *Mental Health*, *Qualitative Research*, *Stigma Barriers*

## Abstract

**Objective:** Many people who access mental health services usually do not seek treatment to avoid the consequences of stigma and label of mental illness. Thus, determining each aspect related to stigma reduction barriers seems necessary.

This qualitative study was conducted to investigate stigma reduction barriers towards mental disorders in Iran.

**Method:** In this study that was conducted from 2013 to 2015, content analysis was used and all stakeholders were selected by purposive sampling technique. All data were obtained through 16 individual interviews, 2 focus groups, and 6 written narratives. The data were collected, coded, and analyzed accordingly.

**Results:** The major themes were as follow: The universality of stigma, beliefs, attitudes and lack of awareness, mental health providers and other specialists, cultural barriers, structures and policymakers, and insufficient financial resources.

**Conclusion:** It is necessarily to identify the barriers of stigma reduction programs in Iran to increase the quality of life of patients with mental disorders. In the present study, due to the presence of mental health stakeholders, the main barriers were obtained.

Many people who have access to mental health services usually do not seek treatment to avoid the consequences of stigma and label of mental illness (1). Psychiatric disorders such as schizophrenia, bipolar, depression and substance abuse increase disability in years of life. Stigma includes approval of prejudicial attitudes, negative emotional responses, biased behavior, and unfair social structures towards a group of people. Stigma not only increases reluctance to help-seeking behavior, it also increases self-stigma and other psychological repercussions (2).

Stigma can be seen in different aspects of human life such as language, disdain in interpersonal relationships and behaviors. It can be considered as a barrier to those patients who need to receive mental health services (3). 

Those patients suffering from mental disorders, usually experience more stigma than patients with other medical conditions. This stigma leads to negative social, economic, political, and psychological consequences. Members of the society are reluctant to interact with these patients. Stigma isolates patients from the society, and as a result, their clinical condition and prognosis will worsen (4). When people do not seek help or delay in seeking help, this can lead to problematic outcomes. For instance, some severe mental illnesses such as psychosis may be worsened. In addition, the period of untreated disorder is related to worse consequences in severe mental disorders such as psychosis, bipolar, major depression, and anxiety disorders(5).

People with severe mental disorders are subjected to discrimination in everyday life situations such as housing, education, and jobs (6). Many studies indicate that people in the United States and Western countries are willing to subscribe stigmatizing attitudes towards mental illness (7). Many researches have been done on public reactions to mentally ill patients (8). The available literature has investigated the adverse consequences of stigma on attitudes of patients and their families, beliefs about mental illness among the public, and strategies to reduce the stigma of mental illnesses, but not the exact mechanism underlying them (9,10,11,12). Knowledge about stigma reduction barriers may be particularly important in providing appropriate health care services and enhancing the quality of life of patients. Therefore, awareness about barriers related to stigma reduction programs in Iran holds great potential for better understanding of this phenomenon. Identifying and understanding each of these aspects related to stigma reduction barriers increases culturally suitable service delivery. Accordingly, a qualitative study was conducted to investigate stigma reduction barriers towards mental disorders in Iran. 

The present study aimed at investigating stigma reduction barriers towards mental disorders in Iran by stakeholders of mental health.

## Materials and Methods

***Design***

This study was conducted in collaboration between academic researchers and stakeholders of mental health. This qualitative study used focus groups, interviews, and content analysis which accomplished in years 2013- to 2015.

***Participants***

Experts of Mental Health, and Addiction office of the Ministry of health as a custodian of mental health assist the researchers to select the stakeholders of mental health. We recruited a purposive sample of 14 stakeholders of mental health (Table 1) and sampling continued to the point of saturation. 

***Data collection and analysis***

At first, individual open interviews were scheduled to collect the data. The researchers were interviewed two 2 times during a three 3- months period with eight 8 of participants. All these interviews facilitated helped the researchers to obtain a deeper understanding towards the phenomenon being investigated. All the interviews were executed in the work place of the participants. The main questions of individual open interviews were consisted as follows: “what is your idea opinion about destigmatization in the field of mental health?”, “what is the role of media in reducing stigma?”, and “what is the barrier of reducing stigma of mental illnesses in Iran?” Each individual open interview took an average of 60 to 90 minutes.

Since the individual interviews did not lead to data saturation, two focus group interviews have been accomplished, which was the main method of data collection of the present study. There were 10 participants in each focus group session. Each focus group session lasted for 120 minutes and was audio -recorded and transcribed verbatim. . Some of the participants participated in both individual open interviews and focused group’s sessions. Thus, overall, 14 participants took part in the study. Focused groups’ sessions were managed by the one of the researchers, and the other researchers accompanied to take notes and made the necessary arrangements. The significant questions of the focused groups were included as follows: “What are your experiences in the field of stigma?”, “What are the major barriers in reducing stigma?”, and “What is the main cause of stigma?”. The consent form was given to all participants to record interviews by a tape recorder. Simultaneously, the field notes, which were recorded immediately after interviews, were reviewed. In total, two focused groups, 8 individual interviews, and 6 written narratives were collected and analyzed. Field notes and unstructured observations were other methods of data collection. Key informants, emerging themes, and data saturation were the main criterion criteria of the researchers about the number of interviews. The researchers carried outperformed data collection, coding and analyzing data at the same time. Constant comparison was applied in all processes of the analysis, and the differences and similarities between the initial codes were distinguished. Same codes were set in a same theme and were conceptualized. All codes were included in a reciprocating motion and they revised if it was necessary. Moreover, all interviews were compared with each other. .All methods which were used in the process of coding s were as follow: comparing the data, asking questions, drawing diagrams, and reviewing notes. MAXQDA software 10.0 R250412 was used to facilitate data analysis and classification. The methods that carried out to increase the precision of the findings were as follow: proper communication with the participants, long-term involvement with the phenomenon (about 24 months), sequential interviews, full immersion in the data, reviewing the findings with participants, overview of observers, utilizing multiple methods for data collection, limited reviewing of literature, detailed recording, and reporting all process of the research.

## Results

Data analysis led to identify 6 main themes and 29 subthemes in participants’ responses about stigma reduction barriers in Iran (Table 2). A. The Universality of Stigma

***A1. Stigma Is Not Limited to Psychiatric Illness*** ***:***

Stigma is extended to cities and different ethnicities and genders (especially women). One of the mental health experts stated, 

Consider this cultural, ethnics and gender oriented jokes which is against women! I do not know what happened and why we should receive many messages and jokes against half of the society members. This is not just against women; it is against our ethnics and our culture as well (P7).

**Table1:** The Demographic Characteristics of the Participants (N = 14)

**Code**	**Sex**	**Organization**	**Position**	**Academic Degree**
P1	Male	Tehran University of Medical Science	psychiatrist/professor	MD
P2	Male	Support Forum of Schizophrenia Patients	Chief/Psychiatrist	MD
P3	Male	Recovered patient	Author and Social activist	BS
P4	Male	Islamic Propagation Organization	Counsellor	MA
P5	Male	Health Office of Tehran Municipality	Employee	MA
P6	Male	Office of Social Pathology Prevention of Welfare Organization	Employee/Psychiatrist	MD
P7	Male	Association of Clinical Psychology	Chief	PhD
P8	Female	A family member of psychotic patient	A member of Support Forum of Schizophrenia patients	MA
P9	Male	University of Social Welfare and Rehabilitation	Professor	MD
P10	Male	Islamic Republic of Broadcasting (IRIB)	Chairman in TV/Psychiatrist	MD
P11	Male	Iranian Health Education and Promotion Association	Chairman	PhD
P12	Female	Office of Social Pathology Prevention of Welfare Organization	Employee/General physician	MD
P13	Male	Armed Forces Medical Services Insurance Organization	Chairman/General physician	MD
P14	Female	Health Insurance of the Ministry of Welfare, Cooperation, Labor and Social Affairs	Employee/General physician	MD

**Table 2:** Stigma Reduction Barriers towards Mental Disorders in Iran

**Categories**	**Subcategories**	**Meaning Units**
**1. Barriers**	**A. Widespread stigma**	Stigma is not limited to psychiatric illness
		global stigma toward mental illness
		Stigma is not limited to psychosis
		The contemporary situation of stigma
		Prevalence of the illness
		Fear and shame resulted from stigma
		Difficulties of psychiatric treatment in comparison to other diseases
		The stigma of psychiatric title
	**B. Beliefs, attitudes and lack of ** **knowledge**	The role of guides and scholars
		Public attitudes
		Public ignorance
		Education of children
		Lack of educational resources for students
	**C. Mental health staff and other ** **specialists**	Negative attitude of psychiatrics and therapists in the field of mental health
		Stigma among physicians (general and specialist) and paramedics
		Impact of illness on patients and families
	**D. Cultural barriers**	Culture
		Media
		Literature and art
		Secrecy and absence of precise statistic
	**E. Structures ** **And policymaker**	Government, ministry of health and inadequacy of cooperation of organizations
		The lack of coherent program
		Problems within an organizational structure
		Psychiatry in not socialized
		NGO

	**F. Insufficient funds**	Insufficient family finances and other financial problems
		Inadequacy of cooperation of donors
		Lack of insurance support
		Budget

**A2.** ***Global Stigma towards Mental Illness***:

There is a global stigma towards mental illness. One of the participants said, 

“Stigma is a global problem. Many impressive stories and movies are releasing all over the world. Besides those movies, in a society if one politician refers to a psychiatrist, he will see its consequences. Recently, one of the American politicians referred to a psychiatrist and it has created many serious media controversy for him (P7).”

***A3.*** ***Stigma Is Not Limited to Psychosis***

Psychotic patients are not only affected by stigma, but also other mentally ill patients such as those with anxiety disorders are affected by stigma. In this case, one participant said, 

 “More than 40 to 50 years ago in Iran psychotic patients were more dysfunctional, and many psychiatrists and psychologists sought for psychotic patients, so people formed a conception about mental illness that if someone refers to a psychiatrist, he/ she has a big irreversible problem (P1).”

***A4. Situation of stigma***

Based on the high rate of mental disorders and those who need consultation, a very few individuals refer to mental health professionals. In this regard, one of the participants said,

“We do not have raw data to present ourselves in the world programs, and it means we are not present in the world studies. We need basic and precise data to do any scientific work (P9).” 

***A5. Fear and Shame Resulted From Stigma***

Fear and shame resulted from stigma decrease the likelihood of treatment seeking and retention, so the families and friends of the patients recommended them to abandon treatment to avoid the repercussions of stigma. In the words of one of the professors:

“We witness stigma among government officials. They attempt to hide their mental disorders and do not receive any professional help. This issue suggests the amount of fear towards stigma (P1).” 

***A6. Treatment Difficulties of Psychiatric Patients***

Physically ill patients refer to a physician without confronting stigma; in contrast, mentally ill patients experience more stigma from people and the government. In this regard, one of the participants said:

“In social communication when people see a blind man in the street usually they do not hesitate to help him, but they avoid mentally ill patients. I think there is more fear towards mentally ill patients than physically ill patients. For example, a patient with cancer is taken to hospital earlier than a mentally ill patient. Actually they do not get any support (P3).” 

***A7. The Stigma of Psychiatrist Title***

The psychiatrist title receives more stigma in comparison with other health specialists. In this regard one of the participants said:

“Many psychiatrists change their title to specialist in mental health on their boards, because if you do not mention this on your board no one would refer to you (p2).”

Or:

“Some patients spent high costs for multiple visits, ineffective imaging, and laboratory procedures. All these efforts are simply because they don’t want to submit psychiatric prescription to their insurance companies, so nobody would suspect that they have mental illness (P 2).”

**B. Beliefs, Attitudes, and Lack of Knowledge**

***B1. The Role of Scholars and Guides in Iran***

 Considering the important role of scholars and guides in Iran, some of them have misleading concepts towards mental disorders. In this regard, one of the participants said,

“Sometimes these scholars and guides pointed out ‘spiritual problems’ instead of mental disorders in their speeches, while many problems are psychiatric and they have nothing to do with spiritual problems. What is this spirit that is mentioning in this country (P9)?”

Another participant who is psychologist and clergyman, said:

“When counseling services were developed in Islamic propagation organization, everyone showed a strong objection. They said we already thought we are employees of the center, but now we think we are insane (P4).” 

***B2. Public Attitudes***

Inappropriate attitudes create public misconception towards mental illness and hospitalization. One of the participants who are a family member of a schizophrenia patient and a social activist said,

“In dominant culture of the society, referring to one of the mental health professionals lead to receiving many labels from acquaintances, friends, and colleagues. In such circumstances, the patient would say: never trouble until trouble troubles you (P8).”

***B3. Public Ignorance***

Public ignorance and inadequate knowledge about mental disorders are crucial barriers of stigma reduction programs. One of the stakeholders said,

“Unfortunately, because of the lack of knowledge, when someone is suffering from mental disorder, their preference is to refer to a fortuneteller or exorcist and after all they decide to refer to a general physician, psychiatrist, or psychologist (P1).”

***B4. Education of Children***

There are inadequate considerations about mental health in children’s education and this is another obstacle of stigma reduction in the society.

“We have failed to declare mental health issues in children’s education. In addition, we do not emphasize that mental health is as important as physical health. Children do not learn anything about mental health; instead, they learn the word crazy (P5).”

***B5. Lack of Educational Resources for Students***

Students have a crucial role in the society, however, there is no specific course in the field of mental health in their Curriculum. 

“It has been 10 to 15 years that we have been striving to provide a 2- unit course about mental health issues for all students, but the authorities at the Ministry of Science do not accept to do it (P7).”

***C. Mental Health Providers and Other Specialists***

One of the serious barriers in reducing stigma in Iran is the ineffective role of therapists and other professionals in the field of mental health.

***C1. Diverse Attitudes of Psychiatrists and Psychologists towards Mental Health Issues***

Psychiatrists and psychologists do not have common understanding towards mental health issues. Another barrier of stigma reduction can be the involvement of non-experts in the field of mental health. 

“We have to emphasize on psychological services such as counseling and psychotherapy and the governmental and insurance organizations must cover these services as well (P1).” 

***C2. Stigma among Physicians (General and Specialists) and Paramedics***

Unfortunately, there are not adequate educational programs about mental disorders for physicians, thus, many physicians and paramedics develop negative attitudes, which provoke stigma towards mental illness. One of the participants said,

“Sometimes specialists cannot find any physical symptoms and claim that we did all investigations and we could not find anything, now you need to refer to a psychiatrist! (P1).”

***C3. Impact of Illness on Patients and Families***

Long-term treatment of patients with mental disorders and involvement of their families is another problem of these patients, which can perpetuate the stigma. One of the participants who had a close contact with her mentally ill relative said,

“It is stigma when we hardly can tell our children that you need a consultant. If one member of the family suffers from a mental disorder, the others should not talk about it. My niece cannot tell anything about her fathers’ disorder in school. They try to hide it from others. As an aunt, I cannot allow myself to say anything about it to others (P8).”

**D. Cultural Barriers**

***D1. Culture***

Culture is an important factor, which either reduces or increases stigma in every society. Most of the participants agreed that any weakness in cultures could create more stigma. Perfectionism is one of the prominent features of our culture. 

“Perhaps the Iranian perfectionist culture will make it worse. Everything should be perfect and pleasant in this culture. Especially, we do not expect to have any defects (P7).”

***D2. Media***

 Another effective component of culture on stigma is media. One of the factors that causes stigma in the society and confront patients with many difficulties is inadequate and unscientific knowledge in the media. According to one of the participants,

“All Media such as radio, Television, or newspaper and magazines should accept that they have humiliating attitudes towards mentally ill patients. They insult mentally ill patients (P11).” 

***D3. Literature and Art***

Recently, artists and writer’s works and speeches are full of inaccurate components about mental disorders. Words such as “mental”, “mad”, and “insane” are common words in our literary culture. One of the participants said,

“I worry about artists and writers in our society. A few days ago, a program was broadcasting on television, which was interpreting the strange sexual behavior of a shrink as a schizophrenic behavior. Wasn’t it possible to use another term for this type of behavior?(P2).”

***D4. Secrecy and Absence of Precise Statistics***

Some officials insist on concealing the true prevalence of mental illnesses, which is one of the most important barriers in understanding the problems of mentally ill patients. One of the participants, who was in the recovery phase of schizophrenia stated, 

“My family asked me not to interview with any medias because they want to hide my disorder from our relatives and other acquaintances”, and

“My family hides my disorder from neighbors, our relatives, and acquaintances. However, always there is a question that why I am not working or why I am not getting married? (P3)”;

 “To receive an invitation to participate in this meeting, they asked me to give a fax number, I was thinking to give one of my relatives fax number, but it came to my mind that they may figure out about the theme of this meeting (P3).”

*Another participant said* *,*

“In my opinion, the authorities are hiding statistics and studies deliberately because when statistics are revealed awareness towards mental illness will increase and we will witness the growth of demands in the society (P8).”

***D5. Unknown and Prominent Under control Patients***

Unknown improved patients bring it to our mind that mental illness is not controllable and this group of people can never have a successful life.

“However, many mentally ill patients are famous and we should introduce them to the society like John Nash, who was awarded the Nobel Prize or many other unknown patients in our country that have a successful life (P7).”

**E. Structures and Policymakers**

***E1. Government, the Ministry of Health, and Inadequate Cooperation of Organizations***

Inappropriate cooperation among scientific centers is another barrier of stigma reduction. In this study, we have heard these types of sentences:

“There is no special position for custodian organizations of mental health. Lack of integration among organizations in the field of mental health can be considered as an important problem. For example, the small part of Ministry of Health (an office) is assigned to mental health and it includes 3 parts: psychiatric, addiction, and social problems, which is not enough for a whole country (P5).” 

***E2. The Lack of Coherent Program***

The absence of a coherent program and lack of organized activities in the country leads to lack of proper solutions. According to one of the participants,

“Important factors in increasing stigma are as follow: mental health is unidentified, there is no specific custodian organization for mental health, and there is no integration among custodian organizations (P5)

***E3. Problems within an Organizational Structure***

According to the complex structures of cities and the prevalence of mental illness, difficulties will be increased and the weakness in structures can be easily observed. In this regard, one of the participants, who had the experience of working on chronic mental illness, expressed:

“According to the circumstances, it seems that the relationship between health and social structures is disconnected. We have to increase our demands and go beyond mere awareness and then observe this awareness in structures and relations (P6).” 

***E4. Psychiatry is not Socialized***

Major problems in promoting mental health in Iran are as follows: mental health is not introduced, psychiatry is not socialized, the stigma of being a psychologist and psychiatrist, mental health is limited in clinics, and the custodian of mental health is not specified. One of the participants said, 

“This filed is not introduced by physicians in the society. We could not introduce mental health in the society (P5)”.

“Another important thing is that psychiatry is not socialized. We have not had socialized many other fields, and this is a problem in the field of health. Nowadays, mental health is presented only in clinics and policlinics (P6).”

***E5. Non-governmental Associations (NGO)***

Other mental health problems are the small number of NGOs, lack of attention of authorities, and lack of executive power in organizations. Very few NGOs are working in the field of mental health and they do not receive enough attention. They do not have actual power and are not efficient for patients (P6).”

“Patients with diabetes or kidney disease refer to NGOs and receive support and facilities, so they will be encouraged to be covered by the NGOs (P8).”

**F. Insufficient Funds**

***F1. Insufficient Financial Family Resources and other Financial Problems***

In today’s world, financial resources play an important role in reducing stigma and giving services. The important problem of families is inadequate financial resources. A participant stated, 

“The most central issue of stigma is budget because the economic difficulties of families and medical expenses are too high (P8).”

***F2. Inadequacy of Donor’s Cooperation***

Donors and charities are a central component of health services in Iran. However, in the field of mental health they are not willing to help.

“When donors are notified about a mental disorder, they do not want to help and say mental patients don’t need any help! (P2).”

***F3. Lack of Insurance Support***

The major concerns of patients and their families are expenses of long-term treatment by insurance organizations.

“Patients feel bad because insurance organizations do not cover them like many other patients with special diseases. If these patients can be covered by special insurance, the Social Security Organization will be responsible and this may help reduce stigma (P8).”

***F4. Budget***

Appropriate funding to reduce or modify a problem shows its importance to the custodians. 

“Authorities are consciously hiding statistics. They also try to hide demands because when the statistics are revealed, awareness in the society will increase and demands will become greater. After all, patients will be identified and services should be provided for them (P8).”

## Limitations

One limitation of the present study was the absence of representatives of some institutions and organizations. Another limitation was the lack of review of literature in Iran. It is advised that future qualitative and quantitative research obtain a precise perspective about barriers of stigma. 

## Discussion

The results of the data analysis resulted in 7 main categories which were as follow: (1) the universality of stigma; (2) beliefs, attitudes, and lack of awareness; (4) mental health providers and other specialists; (5) cultural barriers; (6) structures and policymakers; and (7) insufficient financial resources.

The present study found that attitudes and beliefs play a fundamental role in acceptance of mental illness. These attitudes are affected by the stigma in the society. Stigma and discrimination have an undeniable effect on promoting mental health in every society. Various studies have supported these findings (13, 14,15,16).

A study by Fiorillo et al. (2013) in 27 countries and 108 European organizations showed that stigma is a main therapeutic priority for psychiatrists, mental health professionals, health authorities, and non-governmental organizations that are related to mental health. In the present study, all participants agreed that stigma and discrimination should be one of the priorities of mental health programs in the country. 

All participants of the study pointed out that all aspects of psychiatry are affected by stigma.

The present study like many other studies showed that not only neurotic and psychotic patients are suffering from stigma, but also their families tolerate it (17,18,19).

The present study obtained the same results as Alonso et al (20). They found that in addition to schizophrenia and bipolar patients, other mentally ill patients are affected by stigma. Moreover, similar to Sayarifar et al. (21) participants of the present study emphasized that stigma is an inhibitory factor for depressed patients in requesting help.

Psychiatric treatments, especially medications, ECT and hospitalizations, are affected by stigma and other degrading labels (22,23,24,25,26,27). Five participants considered the present study as one of the first steps in fighting against stigma in the country. One of the barriers from participant’s point of view was involvement of non-specialists such as fortunetellers and exorcists. These non-specialists abuse patients and cerate many difficulties in the course of treatment. Referring to traditional therapists has been mentioned in a study by Akighir et al. (28) and Alem et al. (29) These studies showed that people rely on traditional therapists more that physicians and psychiatrists. Participants believed that culture of perfectionism in Iran could intensify the effects of stigma. Perhaps, because of this point of view there is secrecy in revealing statistics. The tendency to hide mental disorder and conceal statistics by some authorities has resulted from cultural characteristics. In the present study, many participants mentioned cultural weaknesses as barriers of stigma reduction. Furthermore, media as a cultural representation of society do not have enough knowledge in the field of mental health, creating a negative image of mental health. Other qualitative researches in Iran observed that about one third of families tried to hide their disorder from others (30,31).

Most of participants agreed that media shows an unscientific and negative image towards patients. The media should consult with mental health experts especially in programs, which discuss mental health. Literature and art works are full of stigmatizing indications. In many of these works, a mentally ill patient is defined as antisocial and aggressive person. Weakness of governmental structures is another main barrier for stigma reduction. The Ministry of Health and Medical Education, as a custodian of mental health has allocated a small office to address mental health issues in all over the country. Moreover, it does not have enough executive power as a custodian. It seems that among other problems in the field of health, mental health is not a priority issue; in addition, there is not sufficient coordination among different organizations. Therapeutic services would not satisfy patients. There is a distance between society and therapeutic structures, which can increase stigma (32).

Participants in the present study focused their attention on the role of attitudes and beliefs towards stigma in the society. Public opinion towards mental health problems is associated with fear and labels. This point of view will be transferred to children through media and people. Thus, the mentality of children towards mental illness will be negative and degrading (33)

Even some mental health providers who have worked in the field of mental health for many years are affected by these negative attitudes. The therapeutic team, policymakers, and authorities have intensified the negative consequences of stigma (34). Another recommendation of participants was changing the available therapeutic structure to reduce stigma. The new structures would give importance to mentally ill patients, financial needs, relationship among therapeutic team, and therapeutic alliance. Aviram et al. (35) also emphasized the interpersonal relationship between patients and therapists.

During the present study, we noticed that policymakers and planners do not have any comprehensive plan to reduce stigma. The roots of this discrimination trace back to negative attitudes of policymakers. Furthermore, some policymakers try to conceal the precise statistics. Naturally, lack of information and statistic causes to ignore the role of psychiatry in strategic planning. 

Budget and financial issues was one of the most important barriers. In some cases, even this limited budget was not given to mental health providers. The budget planners are affected by stigma and negative attitudes; and as a result, the appropriate budget is not allocated to mental health issues. Moreover, it was stated that patients and their families have many difficulties to cover their financial needs.

All participants in the present study agreed that there is stigma and discrimination among medical staff including physicians, other specialists, and psychiatrists. Participants were not hopeful about changing attitudes among older physicians through available educations, but they were hopeful about changing attitudes and beliefs of students through proper education. In this regard, some changes in educational curriculum should be considered. Obviously, educational programs for this group of people would be more scientific. Unfortunately, like many other studies, this study found that medical students achieve their information about mentally ill patients through media specially movies, not from scientific literature. Therefore, most studies in Iran and other places reveal a negative attitude among students towards mental patients (36,37,38).

According to the important role of clergymen in different aspects of the society, participants agreed that their beliefs and opinions could be effective in shaping attitudes of people. Therefore, it was emphasized to have joint programs with this influential and powerful group. Islamic Propagation Organization, considering the possession of nearly 2000 missionaries in various parts of the country, has announced its eagerness to participate in reducing stigma programs. Findings of the present study showed the barriers of stigma in Iran, which was similar to many other studies.

## Conclusion

In a nutshell, the present study is an initial step in understanding the stigma barriers of mental health in Iran and has crucial implications for the development of destigmatization interventions and necessary actions. According to the stakeholders of the present study, beliefs, attitudes and lack of awareness was one of the most important factor. It is recommended to accomplish further studies to explore more data about the barriers of stigma in Iran. 
